# Comparison of clinical manifestation of dengue fever in Bangladesh: an observation over a decade

**DOI:** 10.1186/s12879-021-06788-z

**Published:** 2021-10-29

**Authors:** Mohammad Jahid Hasan, Tamanna Tabassum, Mohiuddin Sharif, Md. Abdullah Saeed Khan, Akhi Roy Bipasha, Ariful Basher, Mohammad Rafiqul Islam, Mohammad Robed Amin

**Affiliations:** 1 Pi Research Consultancy Center, Dhaka, Bangladesh; 2 Department of Medicine, Dhaka Medical College and Hospital-2, Dhaka, Bangladesh; 3 BSMMU, OSD, Directorate General of Health Service, Dhaka, Bangladesh; 4 Department of Medicine, Shaheed Suhrawardy Medical College, Dhaka, Bangladesh

**Keywords:** Dengue outbreak, Shift of paradigm, Dengue fever, Dengue hemorrhagic fever, Dengue shock syndrome

## Abstract

**Background:**

The clinical presentation of dengue fever had been observed to change with time since its first outbreak in 2000 in Bangladesh. This report showed the clinical presentation of the 2019 outbreak in Bangladesh along with its comparison to previous outbreaks witnessed in this region.

**Methods:**

This hospital-based cross-sectional study was conducted in one of the largest tertiary care hospitals in Dhaka city. A total of 553 laboratory-confirmed and 194 probable dengue cases were interviewed. The clinical manifestation of the confirmed cases of the current outbreak was compared with three of the outbreak reports retrieved from the databases. R version 3.6.3 was used for data analysis.

**Results:**

Among the confirmed cases, two-thirds were male (63.2%) and the average age was 27(± 11) years. Positive tests for NS1 and IgM were present in 99.6% (n = 525/527) and 82.6% (n = 38/46) of the cases, respectively. Thrombocytopenia was present in 66.1% of cases. Fever (100%) was common for all. Gastrointestinal (GIT) features, including abdominal pain (86.5%), anorexia and/or vomiting (69.6%), and Diarrhea (> 3 motions/day) (26.2%) were more frequent than typical rash and other pain symptoms. Hypotension was present in approximately a quarter of patients (25%). GIT features (anorexia, nausea, and/or vomiting) and hypotension were more common among adult participants while bleeding manifestation (melena and vaginal bleeding, p = 0.009 & 0.032) was more frequent in pediatric patients. Compared to outbreaks of 2008, 2016, and 2018, increasing trends in GIT symptoms e.g. anorexia, abdominal pain, and diarrhea were observed. While a negative trend in hemorrhagic manifestations (skin rash, melena, and conjunctival hemorrhage/hemorrhagic sclera) and arthralgia/joint pain were found.

**Conclusion:**

The present outbreak was noticeably characterized by GIT symptoms and hypotension in addition to the typical clinical features like rash and pain symptoms. An increasing trend in GIT features and decreasing trend in hemorrhagic manifestations was noted over the last decade of dengue outbreaks.

**Supplementary Information:**

The online version contains supplementary material available at 10.1186/s12879-021-06788-z.

## Background

Dengue fever (DF) is an arthropod-borne viral disease that affects over 100 million cases worldwide [1, 2]. Bangladesh is a country that is most adversely affected by dengue almost every year [3]. The first official outbreak of dengue was reported in 2000 with a total of 5551 cases and 93 reported deaths [4]. Since then, several incidents of the outbreak were observed over the decade. An estimate suggested the reported dengue cases were 2430 (in 2001), 6232 (in 2002), 3934 (in 2004), 3162 (in 2015), 6060 (in 2016), and 10,148 (in 2018) [3]. In 2019, the country witnessed its largest outbreak in history with > 100,000 confirmed cases and 120 deaths [5]. In 2020, amidst the pandemic, a total of 1026 confirmed cases were reported by the Directorate General of Health in Bangladesh [6].

The clinical manifestations of dengue infection range from mild febrile illness (i.e., DF) to severe hemorrhagic disease (i.e., DHF) and Dengue Shock Syndrome (DSS) [7, 8]. Classical dengue patients usually present with fever, arthralgia, myalgia, retro-orbital pain, and rash. Dengue cases also present with hemorrhagic manifestation (e.g. sub-conjunctival hemorrhage, petechiae, epistaxis, etc.) with or without shock [7]. Respiratory symptoms, gastrointestinal disorders, reduced platelet count, and abnormal liver function tests were also evident as presenting features of dengue now a day [9]. A temporal variation in the frequency of different clinical manifestations can be noted over the decade since the first outbreak [10–14] For example the outbreak of 2000 and 2002 were characterized by high-grade fever with typical purpuric rash, break-bone body ache, and thrombocytopenia [13, 15], whereas in 2010 [12] and 2018 [11] outbreaks, predominant manifestations were fever, gastrointestinal symptoms and bleeding manifestation with normal platelet counts. Frequent transitions to plasma leakage leading to respiratory distress syndrome and organ dysfunction were more commonly observed and were considered a predicting factor of higher case fatality [12, 13]. A few other reports agreed with the preceding result [16, 17]. Shifting of serotypes (DEN-1, 2, 3, and 4) across the outbreaks and re-infection were hypothesized as an underlying cause of this change in clinical manifestation [18, 19]. Detailed serotype data in the individual outbreak are currently lacking. However, DEN-3 has isolated the outbreaks before 2002 [1, 8], while DEN-1 and 2 were the prevalent strain in the outbreaks between 2013 and 2016 [18]. Re-emergence of the DEN-3 was noticed in 2017 and DEN-3 and DEN-4 were more commonly found in recent times [19]. Even, co-detection of dengue serotypes in multiple combinations (DEN-2 & DEN-3 or DEN-1 & DEN-3 or DEN-1, 2 & 3) were observed in several cases in the last two outbreaks. Cross-reactivity of the antibodies therefore might be the reason for severe dengue infection as well as the changed pattern of clinical presentation of the dengue cases in recent time.

Dengue is an emerging public health problem in Bangladesh. From the clinical point of view, identification of shifts in the clinical pattern will improve case-finding and clinical management. Thus, this study aimed to give an insight into the shifting of clinical manifestation of dengue cases in Bangladesh.

## Methods

Here, we initially performed a cross-sectional analysis of the clinical manifestations of dengue during the period of July 2019 to December 2019. Subsequently, we compared these findings with the findings of those studies that were undertaken in similar hospital settings and which included similar types of patients. This was done with the objective of analyzing the changes of the clinical manifestations of the dengue over different time periods.

The initial descriptive cross-sectional study was conducted at the inpatient Department of Medicine, ‘Dengue Corner’ of Sheikh Hasina Burn and Plastic Surgery Institute and Department of Pediatrics in Dhaka Medical College Hospital (DMCH) during the dengue outbreak of 2019. Sample size was calculated from the following formula: $n=\frac{{z}^{2}p(1-p)}{{d}^{2}}$, where, z = standard normal deviate, set at 1.96 at for 95% confidence level, p = estimated prevalence of infection, and d = margin of error (0.05). Considering unknown (50%) prevalence and using 5% margin of error, our calculated sample size was 385. However, we included 747 patients in this study. Details of the methods are given below. The study adheres to the STROBE guideline for cross-sectional study and available in Additional file 1: Table 1.

To compare the clinical manifestation of different dengue outbreaks over the last two decades, we sought studies having three criteria: (i) conducted in the same settings (DMCH), (ii) study followed same study designs (cross-sectional), and (iii) reported dengue outbreak of Bangladesh other than 2019. Four databases (PubMed, BanglaJol, Scielo, and Google Scholar) were searched to find out outbreak reports with keywords ‘Dengue’ AND ‘Bangladesh’. Bibliographic search and personal communication were also used to find out published and unpublished works which documented previous outbreaks within the periods of the years 2000–2019. Database searches were limited up to December 2019. Details of the searching strategy are available in Additional file 1: Fig. 1. Studies that were conducted in Dhaka Medical College Hospital and reported an outbreak of dengue in the different periods were selected for comparison. Finally, three original articles that fulfilled the criteria (i.e. Outbreak report of 2008 by Arif et al. [13], 2016 by Pervin et al. [20]; 2018 by Sultana et al. [21]) were used for data extraction and comparison with the latest outbreak of 2019. The collective variables data from each of the above-mentioned reports were compared, specifically for differences in clinical manifestations. The study characteristics are described in Additional file 1: Table 2. Details of statistical comparison are mentioned in the statistical analyses section.

### The rationale of study site selection

Dhaka Medical college hospital, situated at 23.7257° N, and 90.3971° E, [22] is one of the largest tertiary care hospitals situated in the capital of Dhaka city. According to the Health bulletin published by the Directorate general of health services, the hospital dealt with 935,876 outpatient visits, 172,343 inpatients admission, and 462,438 emergency ward attendants in the year 2017 [23]. The hospital provided care for various health problems for adults, children, and the geriatric population. In the period of outbreak, it operates a dengue corner equipped with a multidisciplinary team specialized in the management of dengue during the outbreak. As a result, the hospital receives referrals and admission from nearby districts and cities of the country. In addition, the government pays a subsidy to keep the centers going. Hence, the center is almost always considered the prime referral center as well as a nationally representative hospital of the country [24].

### Procedure of data collection in 2019 dengue outbreak

Clinically suspected cases of dengue were initially approached for inclusion. A person living in or traveling to the dengue-endemic zone with fever along with any two of the following symptoms—nausea/or vomiting, rash, aches and pains, positive tourniquet test, leucopenia (< 4000/mm^3^), and any warning signs (abdominal pain/tenderness, persistent vomiting, clinical fluid accumulation, mucosal bleed, lethargy/restlessness, liver enlargement > 2 cm, increase in hematocrit with a rapid decrease in platelet count)—was considered a probable dengue case. On the other hand, characteristics of the probable dengue cases along with a positive NS1 antigen (Ag) and/or anti-dengue IgM test were referred to as confirmed dengue cases. This approach was based on the guidance of ‘The national guideline for clinical management of Dengue Syndrome, Bangladesh 2018’ [25]. Written informed consent was ensured before participation and. In case of minors, written consent was taken from the parents or legal guardians of the parents. The study population was free to give information during the study and no benefits were offered for participation. Patients not providing consent or having co-infection were also excluded from the study. A detailed case record form was prepared for data collection and used throughout the data collection period. Demographic information e.g. age, gender, residence, occupation was collected. Clinical characteristics and laboratory investigations were also sought and recorded. Complete blood count (CBC) was routinely evaluated for all patients with an automated hematology analyzer, Mindray|BC-5150. Leukopenia was defined as a total WBC count of < 4000/mm^3^. Thrombocytopenia was defined as a total platelet count of < 100,000/mm^3^. For the detection of non-structural viral protein (NS1 Ag) commercially available kits (Rapid test kit, In Bios Int USA) were used. The IgM antibody and IgG antibody detection in blood samples were carried out using a commercially available kit supplied by DRG International (USA). Raised alanine aminotransferase (ALT) and aspartate aminotransferase (AST) were defined as > 50 IU/L in both cases. Raised bilirubin was considered when values raised > 2 mg/dl. Selecta PRO-M auto analyzer was used to estimate serum bilirubin, ALT, and AST. All laboratory investigation was performed in the laboratory of the DMCH. However, blood tests were requested based on the condition of the patients assessed by study physicians and differed from case to case. Clinical information was collected from the history and physical examination of the patients. Record review was done to collect the report of the investigations. A total of 793 cases were approached, and 36 patients were excluded due to not providing consent, or having coinfection or critical illness. Furthermore, 10 cases were excluded due to incomplete data (> 50% of variables missing information). More details are described in Additional file 1: Fig. 2. Finally, a total of 747 cases (553 confirmed and 194 probable dengue cases) were interviewed. In this study, only confirmed dengue cases are described and used for comparison.

### Ethics statement

The study protocol was reviewed and received approval by the Ethical Review Committee (ERC) of Dhaka Medical College (DMC) in 2019 with registration no: MEU-DMC/ECC/2019/251. The outbreak reports used to compare the clinical characteristics of dengue patients were retrieved from the database, hence waived for ethical review.

### Data extraction for comparison of the outbreak

We extracted the proportion of available symptoms from selected outbreak reports. Following extraction, we tabulated the information using a Microsoft Excel spreadsheet.

### Statistical analyses

Collected data were sorted and cleaned with necessary modifications. Missing values were managed by subtracting the data from the final data set. No mean imputation was made. To describe the characteristics of the confirmed dengue cases of the 2019 outbreak, we used mean ± standard deviation, and percentage when appropriate. Differences in clinical characteristics between adults and children were compared using the Pearson Chi-square (χ^2^) or Fisher’s exact test for categorical variables, or the Student *t*-test or the Mann–Whitney U test for continuous variables. Changing patterns of each symptom over the decade were tested by Cochran–Armitage-test. The proportion of different outbreaks were considered. Here, symptoms reported by at least 3 outbreaks at any point in the period were considered for trend analysis. A negative slope indicates the decreasing proportion of symptoms and a positive slope indicates a positive linear trend and explains the increasing symptoms over the period. For statistical testing, statistical software R version 3.6.3 was used, and p-values of < 0.05 were considered statistically significant.

## Results

A total of 747 suspected dengue cases were approached and 553 cases were confirmed by laboratory investigation and 194 were designated as probable cases. Differences between the confirmed and probable dengue cases are available in Additional file 1: Tables 3 and 4.

Among the confirmed cases (n = 553), 63.2% (347) were male. Predominant age group was 21–30 years (37.7%) [age range 3–85 years]. The mean age of the total population was 27 (± 11) (SD) years and 38.2% reporting a history of travel to an endemic zone (Table 1).

**Table 1:** Demographic characteristics of study population in outbreak 2019

Variables	n (%)
Age (n = 422)	
< 10 years	7 (1.7)
11–20 years	134 (31.8)
21–30 years	159 (37.7)
31–40 years	77 (18.2)
41–50 years	29 (6.9)
51–60 years	9 (2.1)
61–70 years	5 (1.2)
> 70 years	2 (0.5)
Mean (± SD)	27 (± 11)
Sex (n = 549)	
Male	347 (63.2)
Female	202 (36.8)
Occupation (n = 269)	
Student	105 (39)
Housewife	84 (31.2)
Service holder	36 (13.4)
Businessman	26 (9.7)
Farmer	5 (1.9)
Hawker	4 (1.5)
Driver	3 (1.1)
Govt. service	2 (0.7)
Teacher	2 (0.7)
Rickshaw puller	1 (0.4)
Unemployed	1 (0.4)
H/o travelling in endemic zone (n = 453)	173 (38.2)

Decimal number were rounding up with nearest one

Fever was present in all patients (100%). Other common symptoms were abdominal pain (41.6%), nausea/vomiting (69.6%), and severe headache (62.7%). Melena (5.4%) and gum bleeding (3.6%) were the two most common bleeding manifestations observed. Among the presenting signs, the most common were hypotension (25%), low pulse pressure (22.1%), and positive tourniquet test (10.5%). Approximately, 5% of patients showed skin rash during admission. The vital signs of the cohort are shown in Table 2.

**Table 2:** Clinical characteristics of confirmed dengue cases (n = 553)

Presenting symptoms	Total (n = 553) n (%)
Fever	553 (100)
Chill (associated with fever)	52 (9.4)
Shivering (associated with fever)	20 (3.6)
Sweating	123 (22.2)
Severe headache	347 (62.7)
Retroorbital pain	216 (39.1)
Redness of eye	67 (12.1)
Back pain	58 (10.5)
Neck pain	39 (7.1)
Sore throat	9 (1.6)
Rash	25 (4.5)
Joint pain	25 (4.5)
Anorexia	210 (38.0)
Nausea	385 (69.6)
Vomiting	385 (69.6)
Diarrhea (> 3 motions/day)	145 (26.2)
Abdominal pain	230 (41.6)
Cough	30 (5.4)
Respiratory distress	25 (4.5)
Convulsion	1 (0.2)
Decreased urine output	13 (2.4)
**Pattern of bleeding manifestations**	
Blood in stool (melena)	30 (5.4)
Gum bleeding	20 (3.6)
Vaginal bleeding	10 (1.8)
Epistaxis	9 (1.6)
Hematuria	3 (0.5)
**Presenting signs**	
Hypotension	135 (25.0)
Low pulse pressure	60 (22.1)
Tourniquets test positive	12 (10.5)
Skin rash	25 (4.5)
Dehydration	31 (7.8)
Anemia	26 (4.7)
Jaundice	4 (0.72)
Edema	1 (0.18)
Pleural effusion	10 (1.8)
Ascites	7 (1.26)
Splenomegaly	1 (0.18)
Hepatomegaly	3 (0.54)

Vital signs	Mean ± SD
Pulse (/min)	97 ± 11
SBP (mmHg)	93 ± 15
DBP (mmHg)	66 ± 12
Respiratory rate (/min)	18 ± 4
Temperature (°F)	100 ± 1

*SBP* systolic blood pressure; *DBP* diastolic blood pressure

Table 3 presents the investigation findings of the confirmed cases. Out of 527 patients, 525 (99.6%) were found positive for Dengue NS1 antigen, and out of 46 patients, 38 (82.6%) were positive for Dengue IgM antibody. Approximately, 66% had thrombocytopenia, 29% had leucopenia. Serum bilirubin, ALT, and AST were found to be high in 13%, 50%, and 67.4% of patients, respectively.

**Table 3:** Investigation findings of situ population of outbreak 2019

Serological tests	n (%)
NS1 antigen (n = 527)	
Positive	525 (99.6)
Negative	2 (0.4)
Dengue IgM antibody (n = 46)	
Positive	38 (82.6)
Negative	8 (17.4)
Dengue IgG antibody (n = 32)	
Positive	19 (59.4)
Negative	13 (40.6)
HCT (n = 344)	
High (> 48%)	17 (3.9)
Normal	418 (96.1)
Hemoglobin (n = 336)	
Low (< 11 g/dl)	38 (11.3)
Normal	298 (88.7)
Leucopenia (n = 272)	
Present (< 4000/mm^3^)	79 (29)
Absent	193 (71)
Thrombocytopenia (n = 384)	
Present (< 150,000/mm^3^)	254 (66.1)
Absent	130 (33.9)
Serum bilirubin (n = 23)	
High (> 2 mg/dl)	3 (13)
Normal	20 (87)
ALT (n = 146)	
High (> 50 U/L)	73 (50)
Normal	73 (50)
AST (n = 132)	
High (> 50 U/L)	89 (67.4)
Normal	43 (32.6)

There were 131 missing values in the age of the participants. To compare the clinical characteristics between pediatric and adults, we used data from 422 patients (Paediatric-96 vs adult-326). It was observed that gastrointestinal features e.g. anorexia (p = 0.028), nausea, and vomiting (p = 0.029) were more frequent than adults while hypotension (p = 0.003) was more prevalent among adults. Bleeding features e.g. melena (p = 0.009), and vaginal bleeding (0.032) were commonly found in the pediatric population compared to adults. More is described in Table 4.

**Table 4:** Clinical characteristics adult and child dengue cases (n = 422)

Presenting symptoms	Pediatric n (%) (n = 96)	Adult n (%) (n = 326)	p value**
Fever	96(100)	326(100)	1.0
Chill (associated with fever)	14 (14.6)	28 (8.6)	0.118
Shivering (associated with fever)	6 (6.3)	14 (4.3)	0.418
Sweating	26 (27.1)	91 (27.9)	1.0
Severe headache	62 (64)	168 (51.5)	0.016
Retroorbital pain	34 (35.4)	82 (25.2)	0.034
Redness of eye	11 (11.5)	26 (8)	0.306
Back pain	13 (13.5)	32 (9.8)	0.346
Neck pain	11 (11.5)	18 (5.5)	0.063
Sore throat	1(1)	4 (1.2)	1.0
Rash	3 (3.1)	22 (6.7)	0.226
Joint pain	9 (9.4)	16 (4.9)	0.137
Anorexia	35 (36.5)	81 (24.8)	0.028
Nausea	77(80.2)	224 (68.7)	0.029
Vomiting	77(80.2)	224 (68.7)	0.029
Diarrhea (> 3 motions/day)	24 (25)	102 (31.3)	0.256
Abdominal pain	40 (41.6)	122 (37.4)	0.45
Cough	6 (6.3)	13 (4)	0.399
Respiratory distress	2 (2.1)	19 (5.8)	0.184
Convulsion	1(1)	0(0)	0.227
Decreased urine output (n = 16)	4 (4.2)	7(2.1)	0.469
Pattern of bleeding manifestations			
Blood in stool (melena)	12 (12.5)	16 (4.6)	0.009
Gum bleeding	7 (7.3)	10(3.1)	0.077
Vaginal bleeding	5 (5.2)	4 (1.2)	0.032
Epistaxis	4 (4.2)	3 (0.9)	0.50
Hematuria	1 (1)	2 (0.6)	1
Presenting signs			
Hypotension	55 (58.5)	234(74.3)	0.003
Low pulse pressure	39 (40.6)	114 (34.96)	0.306
Tourniquets test positive	2 (2.08)	10 (3.06)	0.726
Skin rash	3 (3.1)	22 (6.7)	0.226
Dehydration	4 (4.16)	24 (7.36)	0.345
Anemia	1 (1.04)	1 (0.306)	1.0
Jaundice	0 (0)	4 (1.22)	0.310
Edema	1 (1.04)	0 (0)	0.234
Pleural effusion	2 (2.08)	6 (1.84)	1.0
Ascites	3 (3.1)	2 (2.08)	0.091
Splenomegaly	0 (0)	1 (1.04)	1.0
Hepatomegaly	0 (0)	1 (1.04)	1.0

Decimal number were rounding up with nearest one

**P-value estimated using chi-square test

A comparison of clinical manifestations among four outbreaks is described in Table 5. The proportion of males was persistently more in all outbreaks but the recent outbreak is characterized by the presence of more female cases than the previous outbreaks. Trend analysis supports contracted male presence over time and it is statistically significant (p = 0.0073, slope: − 0.0552). An increasing trend of abdominal symptoms including anorexia, abdominal pain, and diarrhea was observed in recent outbreaks (p < 0.05 and slope: + ve for all cases) whereas the decreasing trend is noticed in terms of hemorrhagic manifestations including skin rash (p = 0.003, slope: − 0.04), melena (p = 0.5421, slope: − 0.0105) or conjunctival hemorrhage/hemorrhagic sclera (p = 0.4131, slope: − 0.0213) compared to the previous outbreak. Classic symptoms like headache are persistently common in all outbreaks and an increasing trend is visible (p ≤ 0.0001, slope: 0.1261). Significant decline trend is observed in arthralgia/joint pain (p = 0.0002; slope: − 0.0781) over the times while opposite trend is noticed for retro-orbital pain/eye ache are (p = 0.0641, slope: 0.0384).

**Table 5:** Presenting symptoms of the dengue patients in 2019 outbreak with comparison of previous outbreak

	Outbreak 2008	Outbreak 2016	Outbreak 2018	Outbreak 2019	Chi-square statistic	p value*
Lead author	Arif et al. [20]	Pervin et al. [21]	Sultana et al. [22]	Current study		
Sample size	55	40	350	553		
Mean age (years)			25	27		
Sex: (%)						
Male	83.63	62.5	68.57	63.2	7.1892	0.0073^β^
Female	16.37	37.5	31.43	36.8		
Clinical features (%)						
Fever		100	100	100		
Pain pattern						
Headache	32.72	25	61.14	62.7	32.0649	< 0.0001^α^
Myalgia/body ache		12.5	44			
Low back pain			3.14	10.5		
Retro-orbital pain/eye ache	16.38	50	20.29	39.1	3.4292	0.0641^£^
Arthralgia/joint pain	9.09	87.5	23.14	4.5	14.2545	0.0002^β^
Neck pain				7.1		
Respiratory symptoms						
Respiratory distress				4.5		
Cough				5.4		
Abdominal symptoms						
Anorexia	5.45	27.5		38	29.3381	< 0.0001^α^
Nausea		27.5		69.6		
Vomiting	40	27.5				
Diarrhoea	3.63	25	5.14	26.2	8.9826	0.0027^α^
Abdominal distension						
Abdominal pain	1.81	30	32.86	41.6	143.39	< 0.001^α^
Clinical signs						
Pleural effusion				2.5		
Ascites				1.8		
Jaundice		12.5		1		
Skin rash		12.5	6	4.5	4.5205	0.0335^β^
Diff. types of hemorrhagic manifestation						
Positive tourniquet test	43.63			10.5		
Melena		7.5	6.1	5.4	0.3717	0.5421^€^
Gum bleeding				3.6		
Haematuria				0.5		
Conj. Hge./hemorrhagic sclera	16.36	20		12.1	0.6699	0.4131^€^
Haematemesis						
Haemoptysis						
Epistaxis				1.6		
Vaginal bleeding				1.8		
Others				2.4		
Less urine output				0.2		
Hypotension	70.9			25		
Convulsion						
Joint swelling						
Lethargy		30				

Statistical analysis was performed for those variables who had data at least for 3 outbreak

*p-value was estimated by using Cochran–Armitage-test and p-value < 0.05 was considered statistically significant

^α^Increasing and statistically significant trend present

^β^Decreasing and statistically significant trend present

^£^Increasing and statistically non-significant trend present

^€^Decreasing and statistically non-significant trend present

## Discussion

Bangladesh is frequently facing outbreaks of dengue since its emergence in 2000. In 2019 the country faced its worst outbreak in history with infection reaching all parts of the country [26]. Our study revealed that affected persons were mostly young and adolescent males. A characteristic presence of gastrointestinal symptoms alongside the usual general features was a notable finding of this outbreak.

Dengue patients typically present with the triad of fever, pain, and rash. However, gastrointestinal and bleeding manifestations might occur in variable proportions [27]. Previously, a severe form of infection was less frequent than the recent outbreaks in Bangladesh. This change is evident since 2018 [19] when the frequency of severe dengue cases increased. Shirin et al. proposed that a re-infection with DEN-3 serotype on the top of previously predominant DEN-1 and DEN-2 serotypes might be responsible for this increased severity [19] and was later confirmed by Ahsan et al. [28]. Consequently, the spectrum of clinical presentation of dengue might be facing a shift. This study aimed to explore this phenomenon.

Our exploration revealed that the 2019 outbreak was characterized by fever in all patients with the most common associated features being nausea and/or vomiting, severe headache, anorexia, and retro-orbital pain. These are the general features of dengue fever [25] and are well correlated with other similar studies [29–31]. But this year hemorrhagic presentation was relatively less common with approximately 5% having one or more bleeding manifestations. A study from a non-endemic zone of Bangladesh during the 2019 outbreak also reveals similar findings [32]. Our comparison of studies conducted in DMCH in 2008 [20], 2016 [21], 2018 [22], and the current study also reveals a decreasing proportion of bleeding manifestations (e.g., melena). Melena was the most common form of bleeding followed by gum bleeding in the current study. Moreover, skin rash was one of the prime features in previous outbreaks with a statistically significant decrease in recent outbreaks. In contrast studies in India showed a high prevalence of rash in 2015 [33] and 2018 [34, 35].

Another notable finding was a statistically increased frequency of diarrhea and abdominal pain over the last decade. Yung et al. [36] in Singapore observed that abdominal pain was more likely to occur in DEN-3 serotypes than DEN-1 and DEN-2. As the DEN-3 serotype was more prevalent in 2018 and 2019 outbreaks in Bangladesh [28], an increase in abdominal pain could be explained which, however, needs further verification through extensive serotype-specific clinical studies.

In the present study, the most common clinical sign was hypotension (25.4%, n = 135) and low pulse pressure (22.1%, n = 60). A comparison of outbreaks concerning hypotension and pulse pressure was not possible due to the unavailability of data. But these indicators of plasma leakage show that at least one-quarter of the patients may have had dengue shock syndrome [37] in the current study. Interestingly this rapid development of hypotension and shock was not observed in previous outbreaks unlike in 2019, and it probably indicates a shift in severity of the disease.

As years were passing in Bangladesh, we got accustomed to a seasonal upsurge of a disease that seemed very benign at first with an occasional increase in severity. But the obvious change in presentation since the 2018 outbreak was marked by the increased number of cases and fatalities. As the clinical features are shifting physicians must be adept to learn about newer patterns. The epidemiological, environmental, virological, and vector dynamics are playing a pivotal role in this kind of shift. Hence besides clinical understanding, it would be prudent to go for studies related to virus, vector, environment, economics, and epidemiology.

During the dengue outbreaks from 2000 to 2019 patients with dengue, syndrome showed the varied presentation and the symptoms were nonspecific. Dengue virus interacts with host cells and causes the release of various cytokines and stimulates immunologic mechanisms, vascular endothelial changes, infiltration of mononuclear cells, and perivascular edema. Ascites, pleural effusion, and gall bladder edema from capillary leak syndrome are some of those features. But various atypical manifestations were also being added along the passage of time. As a result, Dengue viral infection has become a major public health issue in Bangladesh which is facing dengue outbreaks every year and mortality morbidity due to dengue is rising to an alarmingly high rate with each outbreak.

### Limitation of the study

A major limitation of the study involved missing data due to the inability to systematically filled out by the study physicians. In addition, the overall number of children was small. Moreover, it was not possible to fully investigate all participants and to serotype the dengue cases, mostly due to lack of facility and funds. Lastly, a detailed feature-by-feature comparison of the different outbreaks was not possible due to inadequate details present in the available studies.

### Scope of the study

Despite several limitations, our study was strong in that we reported a large number of cases during the 2019 dengue outbreak in Bangladesh and were able to analyze the changes of clinical manifestations across the outbreaks over time. Therefore, our results may aid in the adaptation and up-gradation of the national guideline and management strategies for future outbreaks in the country.

## Conclusion

A comparison of clinical manifestation of dengue fever in Bangladesh over a decade showed that GIT symptoms were more prevalent than bleeding manifestations in recent times compared to the previous outbreak.

## Supplementary Information

**Additional file 1: Figure 1.** Searching details of the study underwent in DMCH and reporting dengue outbreak in Bangladesh. **Figure 2.** Flow chart of patient selection. **Table 1.** STROBE Statement—Checklist of items that should be included in reports of cross-sectional studies. **Table 2.** Study characteristics that not included for comparison. **Table 3.** Clinical characteristics of confirmed and probable dengue cases* (n = 747). **Table 4.** Laboratory findings of the confirmed and probable dengue cases of the study.

## Data Availability

Data and materials supporting our findings in the manuscript will be shared on reasonable request (dr.jahid61@gmail.com).

## Abbreviations

ALT: Alanine aminotransferase
AST: Aspartate aminotransferase
CBC: Complete blood count
DEN: Dengue serotype
DF: Dengue fever
DHF: Dengue hemorrhagic fever
DMCH: Dhaka Medical College Hospital
DSS: Dengue Shock Syndrome
ERC: Ethical Review Committee
GIT: Gastrointestinal
NS1 Ag: Non-structural viral protein 1
