# Supplementary material for: Comparison of clinical manifestation of dengue fever in Bangladesh: an observation over a decade
Source: BMC Infect Dis. 2021 Oct 29;21:1113. doi: 10.1186/s12879-021-06788-z (PMC8555248; doi:10.1186/s12879-021-06788-z)
Supplement: Supplementary file 1 — Additional file 1: Figure 1. Searching details of the study underwent in DMCH and reporting dengue outbreak in Bangladesh. Figure 2. Flow chart of patient selection. Table 1. STROBE Statement—Checklist of items that should be included in reports of cross-sectional studies. Table 2. Study characteristics that not included for comparison. Table 3. Clinical characteristics of confirmed and probable dengue cases* (n = 747). Table 4. Laboratory findings of the confirmed and probable dengue cases of the study. [file 12879_2021_6788_MOESM1_ESM.docx]

**Additional Figure 1: Searching details of the study underwent in DMCH and reporting dengue outbreak in Bangladesh**

## Included

## Eligibility

## Identification

## Screening

Database Search Results:
Pubmed = 159

Medline (Ovid) = 781

BanglaJol = 111

Google Scholar = 5400

**Total = 6451**

Studies underwent other than DMCH or more than one sites simultaneously
(n = 9)

Study underwent only in DMCH
(n = 3)

Additional records identified through other sources
**(n = 1)**

Records excluded after abstract screening with reason (n = 84)

Records excluded after title screening (n = 6012)

Records screened: titles
(n = 6176)

Records screened: abstracts
(n = 164)

Full-text articles excluded
(n = 68)

Full-text articles assessed for eligibility
(n = 80)

Studies report dengue outbreak since 2000
(n = 12 )

Records after duplicates removed
(n = 276 )

Suspected dengue cases approached
(n = 793)

Case excluded (n=36)
(n=16) Not agreed to provide consent

(n=9) Co-infection: 5 cases of Dengue with typhoid, 3 cases of dengue with malaria, 1 cases of Dengue with HIV

(n=11) Critically ill to participate

Primarily selection of the dengue cases (probable and confirmed)

(n =757)

Cases was excluded (n=10) from analysis due to missing data for >50% variables

Final analysis
(n = 747)

**Additional figure 2. Flow chart of patient selection**

**Additional table 1:** STROBE Statement—Checklist of items that should be included in reports of ***cross-sectional studies***

|  | |  | Item No | Recommendation |
| --- | --- | --- | --- | --- |
| **Title and abstract** | | X | 1 | (*a*) Indicate the study’s design with a commonly used term in the title or the abstract |
|  |  | X |  | (*b*) Provide in the abstract an informative and balanced summary of what was done and what was found |
| Introduction | | | | |
| Background/rationale | | X | 2 | Explain the scientific background and rationale for the investigation being reported |
| Objectives | | X | 3 | State specific objectives, including any pre-specified hypotheses |
| Methods | | | | |
| Study design | | X | 4 | Present key elements of study design early in the paper |
| Setting | | X | 5 | Describe the setting, locations, and relevant dates, including periods of recruitment, exposure, follow-up, and data collection |
| Participants | | X | 6 | (*a*) Give the eligibility criteria, and the sources and methods of selection of participants |
| Variables | | X | 7 | Clearly define all outcomes, exposures, predictors, potential confounders, and effect modifiers. Give diagnostic criteria, if applicable |
| Data sources/ measurement | | X | 8 | For each variable of interest, give sources of data and details of methods of assessment (measurement). Describe comparability of assessment methods if there is more than one group |
| Bias | | X | 9 | Describe any efforts to address potential sources of bias |
| Study size | | X | 10 | Explain how the study size was arrived at |
| Quantitative variables | | X | 11 | Explain how quantitative variables were handled in the analyses. If applicable, describe which groupings were chosen and why |
| Statistical methods | | X | 12 | (*a*) Describe all statistical methods, including those used to control for confounding |
|  |  | X |  | (*b*) Describe any methods used to examine subgroups and interactions |
|  |  | X |  | (*c*) Explain how missing data were addressed |
|  |  | X |  | (*d*) If applicable, describe analytical methods taking account of sampling strategy |
|  |  | NA |  | (*e*) Describe any sensitivity analyses |
|  | Results | | | |
| Participants | | X | 13 | (a) Report numbers of individuals at each stage of study—eg numbers potentially eligible, examined for eligibility, confirmed eligible, included in the study, completing follow-up, and analysed |
|  |  | X |  | (b) Give reasons for non-participation at each stage |
|  |  | X |  | (c) Consider use of a flow diagram |
| Descriptive data | | X | 14 | (a) Give characteristics of study participants (eg demographic, clinical, social) and information on exposures and potential confounders |
|  |  | X |  | (b) Indicate number of participants with missing data for each variable of interest |
| Outcome data | | X | 15 | Report numbers of outcome events or summary measures |
| Main results | | X | 16 | (*a*) Give unadjusted estimates and, if applicable, confounder-adjusted estimates and their precision (eg, 95% confidence interval). Make clear which confounders were adjusted for and why they were included |
|  |  | X |  | (*b*) Report category boundaries when continuous variables were categorized |
|  |  | NA |  | (*c*) If relevant, consider translating estimates of relative risk into absolute risk for a meaningful time period |
| Other analyses | | NA | 17 | Report other analyses done—eg analyses of subgroups and interactions, and sensitivity analyses |
| Discussion | | | | |
| Key results | | X | 18 | Summarise key results with reference to study objectives |
| Limitations | | X | 19 | Discuss limitations of the study, taking into account sources of potential bias or imprecision. Discuss both direction and magnitude of any potential bias |
| Interpretation | | X | 20 | Give a cautious overall interpretation of results considering objectives, limitations, multiplicity of analyses, results from similar studies, and other relevant evidence |
| Generalisability | | X | 21 | Discuss the generalisability (external validity) of the study results |
| Other information | | | | |
| Funding | | X | 22 | Give the source of funding and the role of the funders for the present study and, if applicable, for the original study on which the present article is based |

**NB: X=described in the manuscript**

**Additional table 2:** **Study characteristics that not included for comparison**

|  | **Articles** | **Outbreak report** | **Study site** | **Study design** | **Study population** |
| --- | --- | --- | --- | --- | --- |
|  | Afroze et al.,2002 | 2000 | Bangabandhu  Sheikh Mujib Medical University (BSMMU) | Cross-sectional | Total 31  Children 11  Adult 20 |
|  | Pervin et al., 2004 | 2000 | BSMMU, and Dhaka  Medical College Hospital (DMCH) | Observational | Total 97 (both children and adult) |
|  | Ahmed et al., 2001 | 2000 | Chittagong Medical College Hospital (CMCH) | - | Children 72 |
|  | Islam et al., 2006 | 2002 | Shaheed Shurawrdy Medical College | - | Total 100 (both children and adults) |
|  | Islam et al., 2012 | 2010 | DMCH and popular Medical College Hospital | Observational | Adults 50 |
|  | Islam et al., 2020 | 2011 | BIRDEM General Hospital | Observational | Adults 50 |
|  | RAMAN et al., 2013 | 2012 | Border Guard Hospital, Dhaka | Observational | 200 adults |
|  | Mobarak et al., 2017 | 2016 | Dhaka Sishu Hospital | Observational | Children 56 |
|  | Ahsan et al., 2020 | 2018 | Dhaka Sishu Hospital | Cross-sectional | Children 51 |

| **Additional Table 3. Clinical characteristics of confirmed and probable dengue cases^*^ (N=747)** | | | | | | |
| --- | --- | --- | --- | --- | --- | --- |
| **Presenting symptoms** | **Confirmed dengue**  **(n=553)**  **n (%)*** | **Probable dengue**  **(n=194)**  **n (%)** | | **P value*** | | **Total (n=747)**  **n (%)** |
| Fever | 553 (100) | 194 (100) | |  | | 747 (100) |
| Chill (associated with fever) | 52 (9.4) | 18 (9.3) | | .959 | | 70 (9.4) |
| Shivering (associated with fever) | 20 (3.6) | 4 (2.1) | | .291 | | 24 (3.2) |
| Sweating | 123 (22.2) | 20 (10.3) | | <0.001 | | 143 (18.5) |
| Severe headache | 347 (62.7) | 112 (57.7) | | .217 | | 459 (61.4) |
| Retroorbital pain | 216 (39.1) | 70 (36.1) | | .463 | | 286 (38.3) |
| Redness of eye | 67 (12.1) | 25 (12.9) | | .779 | | 92 (12.3) |
| Back pain | 58 (10.5) | 18 (9.3) | | .631 | | 76 (10.2) |
| Neck pain | 39 (7.1) | 12 (6.2) | | .649 | | 51 (6.8) |
| Sore throat | 9 (1.6) | 1 (0.5) | | .246 | | 10 (1.3) |
| Rash | 25 (4.5) | 10 (5.2) | | .719 | | 35 (4.7) |
| Joint pain | 25 (4.5) | 5 (2.6) | | .236 | | 30 (4.0) |
| Anorexia | 210 (38.0) | 66 (34.0) | | .326 | | 276 (36.9) |
| Nausea and/or Vomiting | 385 (69.6) | 132 (68.0) | | .682 | | 517 (69.2) |
| Diarrhea (> 3 motions/day) | 145 (26.2) | 46 (23.7) | | .491 | | 191 (25.6) |
| Abdominal pain | 230 (41.6) | 66 (34) | | .081 | | 296 (39.62) |
| Cough | 30 (5.4) | 9 (4.6) | | .647 | | 39 (5.2) |
| Respiratory distress | 25 (4.5) | 9 (4.6) | | .837 | | 34 (4.6) |
| Convulsion | 1 (.2) | 1 (.5) | | .438 | | 2 (.3) |
| Decreased urine output | 13 (2.4) | 2 (1.0) | | .259 | | 15 (2) |
| **Pattern of bleeding manifestations** | | |  | |  |  |
| Blood in Stool (Melena) | 30 (5.4) | 13 (6.7) | | .551 | | 43 (5.8) |
| Gum Bleeding | 20 (3.6) | 1 (.5) | | .025 | | 21 (2.8) |
| Vaginal Bleeding | 10 (1.8) | 0 | | .059 | | 10 (1.3) |
| Epistaxis | 9 (1.6) | 0 | | .072 | | 9 (1.2) |
| Hematuria | 3 (.5) | 4 (2.1) | | .059 | | 7 (.9) |
| **Presenting signs** |  |  | |  | |  |
| Hypotension | 135 (25.0) | 48 (24.7) | | .656 | | 183 (24.5) |
| Low pulse pressure | 60 (22.1) | 13 (6.7) | | .032 | | 73 (9.77) |
| Tourniquets test positive | 12 (10.8) | 7 (20.0) | | .142 | | 19 (2.54) |
| Rash | 25 (4.5) | 10 (5.2) | | .719 | | 35 (4.7) |
| Dehydration | 31 (5.6) | 7 (3.6) | | .795 | | 38 (5.1) |
| Anemia | 26 (4.7) | 4 (2.0) | | .359 | | 30 (4.0) |
| Jaundice | 4 (.72) | 2 (1.0) | | .408 | | 6 (.80) |
| Edema | 1 (.18) | 1 (.5) | | .292 | | 2 (0.26) |
| Pleural Effusion | 10 (1.8) | 3 (1.5) | | .724 | | 13 (1.74) |
| Ascites | 7 (1.26) | 5 (2.5) | | .063 | | 12 (1.60) |
| Splenomegaly | 1 (.18) | 4 (2.0) | | <0.001 | | 5 (0.66) |
| Hepatomegaly | 3 (.54) | 2 (1) | | .161 | | 5 (0.66) |
| **Vital signs** |  |  | |  | | **Mean±SD** |
| Pulse rate (/min) | 97±11 | 88±13 | | .150 | | 86 ±13 |
| Systolic Blood Pressure  (mmHg) | 93±15 | 96±16 | | .921 | | 96 ±15 |
| Diastolic Blood Pressure (mmHg) | 66±12 | 66±12 | | .442 | | 66±12 |
| Respiratory rate (/min) | 18±4 | 19±3 | | .564 | | 19 ±4 |
| Temperature (^0^F) | 100±1 | 99.7±1.6 | | .413 | | 99.6±1.4 |

*p value was determined by chi square test, Fisher’s exact test and student t test as appropriate

**Additional** **Table 4. Laboratory findings of the confirmed and probable dengue cases of the study***

| **Serological Tests** | **Confirmed dengue**  **(n=553)**  **n (%)** | **Probable dengue**  **(n=194)**  **n (%)** | **P value^**^** | **Total**  **n (%)** |
| --- | --- | --- | --- | --- |
| **NS1 Antigen (n=571)** |  |  |  |  |
| Positive | 525 (99.6) | 0 | <.001 | 525 (91.9) |
| Negative | 2 (0.4) | 44 (100) |  | 46 (8.1) |
| **Dengue IgM Antibody (n=64)** |  |  |  |  |
| Positive | 38 (82.6) | 0 | <.001 | 38 (59.4) |
| Negative | 8 (17.4) | 18 (100) |  | 26 (40.6) |
| **Dengue IgG Antibody (n=60)** |  |  |  |  |
| Positive | 19 (59.4) | 17 (60.7) | 0.916 | 36 (60) |
| Negative | 13 (40.6) | 11 (39.3) |  | 24 (40) |
| **Hematocrit (n=452)** |  |  |  |  |
| High (>48%) | 15 (4.4) | 2 (2.2) | .344 | 17 (3.9) |
| Normal (≤48%) | 329 (95.6) | 89 (97.8) |  | 435 (96.1) |
| **Hemoglobin (n=484)** |  |  |  |  |
| Low (<11 g/dl) | 38 (11.3) | 11 (12.1) | .836 | 49 (11.5) |
| Normal (≥ 11 g/dl) | 298 (88.7) | 80 (87.9) |  | 378 (88.5) |
| **Leukopenia (n=337)** |  |  |  |  |
| Present (<4000/mm^3^) | 79 (29.0) | 16 (24.6) | .476 | 95 (28.2) |
| Absent (≥ 4000/mm^3^) | 193 (71.0) | 49 (75.4) |  | 242 (71.8) |
| **Thrombocytopenia (n=489)** |  |  |  |  |
| Present (<150,000/mm^3^) | 254 (66.1) | 84 (80.0) | .006 | 338 (69.1) |
| Absent (≥ 150,000/mm^3^) | 130 (33.9) | 21 (20.0) |  | 151 (30.9) |
| **Serum bilirubin (n=30)** |  |  |  |  |
| High (>2 mg/dl) | 3 (13) | 1 (14.3) | .677 | 4 (13.3) |
| Normal (≤ 2mg/dl) | 20 (87) | 6 (85.7) |  | 26 (86.7) |
| **Alanine transaminase (ALT) (n=178)** | | | | |
| High (>50 U/L) | 73 (50.0) | 19 (59.4) | .336 | 92 (51.7) |
| Normal (≤50 U/L) | 73 (50.0) | 13 (40.6) |  | 86 (48.3) |
| **Aspartate transaminase (ASL) (n=168)** | | | | |
| High (>50 U/L) | 89 (67.4) | 25 (69.4) | .818 | 114 (67.9) |
| Normal (≤50 U/L) | 43 (32.6) | 11 (30.6) |  | 54 (32.1) |

*The frequency and percentage presented are after excluding missing values

**P value was determined by chi square test
